# Impact of Processing on Antioxidant Rich Foods

**DOI:** 10.3390/antiox11050797

**Published:** 2022-04-19

**Authors:** Monica Rosa Loizzo, Rosa Tundis

**Affiliations:** Department of Pharmacy, Health and Nutritional Sciences, University of Calabria, 87036 Arcavacata di Rende, Italy; rosa.tundis@unical.it

Food is processed to make it safe, to make its shelf-life more stable, and to make it more desirable [1]. The food processing industry comprises three segments based on levels of processing. Primary food processing includes the collection, classification, and packaging of vegetables, fruits, vegetables, rice, spices, milk, etc. The secondary processing of food includes the actual processing aimed at facilitating its consumption. The segment of tertiary food processing (or functional food) includes fruit and vegetable processing, processing of juices, jams, etc.

Despite the growing worldwide consumption of processed foods, much remains to be understood regarding the exact nature of these changes. This is partly due to the complexity of the reactions involved and partly due to the analytical limits of the methodologies currently available as well as the analysis problems imposed by the intractable nature of the food matrix [2]. Food processing includes treatment, heat treatment by heating or cooling, drying, treatments on the use of pressures, fermentations, etc. 

Processing temperature and time substantially impact food product composition and storage as well as extrusion, fermentation, germination, and milling. Several dietary antioxidants including nutrient and non-nutrient compounds are affected by processing. The most important nutrients with antioxidant properties are vitamins and fatty acids, whereas non-nutrients include polyphenols, flavonoids, carotenoids, etc. [3]. Hence, understanding the impact of processing on the various components of the food is incredibly significant.

We have organized a Special Issue titled “Impact of Processing on Antioxidant Rich Foods” in *Antioxidants* (ISSN 2076-3921; https://www.mdpi.com/journal/antioxidants, accessed on 30 March 2022). This Special Issue reports and discusses the impact of conventional and innovative processing on retaining an adequate level of antioxidants in foods or strategies to limit their loss. This Special Issue includes 16 research papers, 2 reviews, 1 perspective, and 1 communication, all of which are important contributions to this topic by distinguished experts in this area. 

Article 1 is titled “The Different Contributors to Antioxidant Activity in Thermally Dried Flesh and Peel of Astringent Persimmon Fruit” [4]. In this work, authors investigated the impact of thermal-drying processing on persimmon fruit flesh and peel. Thermal drying process reduced the antioxidant activities of the flesh in a temperature-dependent manner. This loss is related to a reduction in carotenoid, phenol, and flavonoid content. At the same time, it enhanced peel antioxidant activities, probably as a consequence of enhanced phenolics and total hydrolysable tannin content. Thus, the application of heat-drying on persimmon peel is a useful process to improve antioxidant activity, aimed at obtaining nutraceutical compounds.

Article 2 (“Effect of Crushing Peanuts on Fatty Acid and Phenolic Bioaccessibility: A Long-Term Study”) [5] is an incredibly interesting study on the consequences of the effects of crushing peanuts on the bioavailability of fatty acids and phenolic compounds in healthy adults. The results of the ARISTOTELE study suggested that the crushing step in peanut butter processing may enhance the bioavailability of fatty acids, which have been associated with protective effects against several chronic diseases. Moreover, the crushing process was found to enhance the phenolic content in the food product, although no differences in phenolic bioavailability were observed. 

In the work titled “Contribution of Hydrolysis and Drying Conditions to Whey Protein Hydrolysate Characteristics and In Vitro Antioxidative Properties” [6], the authors studied the impact of processing on whey proteins (WP). These compounds have demonstrated several health benefits. Enzyme preparation (Alcalase^®^ and Prolyve^®^), used to obtain WP, affected physicochemical and antioxidant activities without any effects on WP powder composition, color, or morphology. The results showed that the pray-dried process is a more suitable technique to obtain WP characterized by greater antioxidant activity than WP obtained using a freeze-drying process.

In “Bioavailability Study of Isothiocyanates and Other Bioactive Compounds of *Brassica oleracea* L. var. *Italica* Boiled or Steamed: Functional Food or Dietary Supplement?” [7], researchers investigated the impact of boiling or steaming processes on the bioavailability of bioactives in broccoli. The steaming process has been demonstrated to enhance isothiocyanates’ plasma bioavailability compared with boiling. However, both treatments led to a similar impact on lutein, β-carotene, and phylloquinone bioavailability. 

Article 5 (“Effect of Deep Frying of Potatoes and Tofu on Thermo-Oxidative Changes of Cold Pressed Rapeseed Oil, Cold Pressed High Oleic Rapeseed Oil and Palm Olein”) [8] explored the thermo-oxidative changes occurring during the deep frying of potatoes and tofu in cold-pressed rapeseed oils and palm olein. The results demonstrated that cold-pressed high oleic rapeseed oil was more stable during deep frying compared with cold pressed rapeseed oil, but much less stable than palm olein. Moreover, frying potatoes affects oil quality deterioration more than frying a protein product such as tofu.

Esposto et al.’s [9] article, “Chemical Composition, Antioxidant Activity, and Sensory Characterization of Commercial Pomegranate Juices”, investigated the effect of high-temperature short-time (HTST) treatment and high-pressure processing (HPP) on the volatile profile of pomegranate juice. 3-Furfural was the most abundant compound in juices subjected to HTST, whereas it was absent in juice treated with HPP. Moreover, heat processing affected the juices’ sensory attributes.

In Article 7 (“Impact of Drying Processes on the Nutritional Composition, Volatile Profile, Phytochemical Content and Bioactivity of *Salicornia ramosissima* J. Woods), the authors demonstrated that compared to freeze-drying, the oven-drying process had a lower impact on the *Salicornia ramosissima* nutritional properties, whereas the phytochemical content and antioxidant activity were significantly lowered [10]. At the same time, neither treatment affected the antiproliferative and antihypertensive properties. 

In “Influence of the Ripening Stage and Extraction Conditions on the Phenolic Fingerprint of ‘Corbella’ Extra-Virgin Olive Oil”, the authors examined the impact of agronomic and technical factors on the phenolic fingerprint of “Corbella” extra virgin olive oil. A negative correlation between the total phenols and higher crushing temperatures was found. The best malaxation conditions were found to occur after 30 min at 37 °C; at the same time, the crushing size did not have any affect [11].

In Article 8, titled “Broa, an Ethnic Maize Bread, as a Source of Phenolic Compounds”, the authors [12] showed that the total phenolic content as well as the content of hydroxycinnamic acids resistant to Portugal bread traditional processing and consequently broa are characterized with promising antioxidant activity. Considering that soluble-free phenolic content increased after bread processing, an enhanced bioaccessibility was observed. 

In “Phenolic Composition and Antioxidant Activity of Purple Sweet Potato (*Ipomoea batatas* (L.) Lam.): Varietal Comparisons and Physical Distribution” [13], the authors investigated the phytochemical content of the purple sweet potato layer that is normally removed during processing. This byproduct represents a possible source of bioactive molecules and of peonidin, cyanidin, and pelargonidin glycosides, as well as quercetin derivatives. Moreover, “Sinjami” and “Jami” cultivars are characterized by a high phenolic content and antioxidant activities.

Article 10 (“Effect of Cooking Methods on the Antioxidant Capacity of Foods of Animal Origin Submitted to In Vitro Digestion-Fermentation”) [14] is a remarkably interesting study focusing on the evaluation of antioxidant activity of different foods of animal origin submitted to different culinary processes and to an in vitro digestion and gut microbial fermentation. Both meat and fish are characterized by interesting antioxidant activity which increased even more after the frying and boiling process. Although foods of animal origin cannot be compared to fruits or vegetables in terms of antioxidants’ phytochemical content, the processes of digestion and fermentation can provide some health properties that could have been previously unappreciated. 

In “Impact of Mild Oven Cooking Treatments on Carotenoids and Tocopherols of Cheddar and Depurple Cauliflower (*Brassica oleracea* L. var. *botrytis*)”, a comparison between steam and sous-vide oven procedures and domestic boiling processes on liposoluble antioxidants of orange and purple cauliflower was made [15]. All the procedures tested resulted in an increase in tocopherols and carotenoids’ extractability. Although the extent of the increase was found to be dependent on the variety of cauliflower, the operating conditions (cooking time and temperature) and the chemical nature of the specific compound did not play a role.

Article 12 (“Effect of Cooking Methods on the Antioxidant Capacity of Plant Foods Submitted to In Vitro Digestion–Fermentation”) [16] described the antioxidant capacity of forty-two different plant foods after in vitro digestion and fermentation. All foods were studied both raw and after grilling, boiling, frying, and roasting. All cooking methods affected the antioxidant activity as well as the release and transformation of antioxidant compounds from the food matrix. In general, the fermented fraction represented up to 80–98% of total antioxidant capacity. 

In “Near UV-Vis and NMR Spectroscopic Methods for Rapid Screening of Antioxidant Molecules in Extra-Virgin Olive Oil”, Vicario et al. [17] applied UV–Vis and NMR to identify and quantify olive oil antioxidant compounds. These compounds strongly contributed to the organoleptic properties of the final product. 

Bonacci et al. [18] in Article 14 (“Natural Deep Eutectic Solvent as Extraction Media for the Main Phenolic Compounds from Olive Oil Processing Wastes”) described the use of Natural Deep Eutectic Solvents (NADESs) for the microwave-assisted extraction (MAE) of valuable bioactive phenolic compounds from olive oil processing wastes. The results indicated that the proposed extraction process is characterized by high efficiency and is eco-friendly. Moreover, NADES extracts could be used in their wholeness, even in products to be used or consumed by people.

In the final article (“*Citrus × clementina* Hort. Juice Enriched with Its By-Products (Peels and Leaves): Chemical Composition, In Vitro Bioactivity, and Impact of Processing”), Leporini et al. [19] proposed a model for the reuse of *Citrus × clementina* by-products for the development of a functional drink characterized by antioxidant, hypoglycemic, and hypolipidemic activities. Even though the pasteurization process reduces the content of bioactives, it did not significantly affect the bioactivity of the by-product-enriched juices.

In the communication (“A Lesson Learnt from Food Chemistry—Elevated Temperature Triggers the Antioxidant Action of Two Edible Isothiocyanates: Erucin and Sulforaphane”) [20], the researchers demonstrated that both sulforaphane (SFN) and erucin (ERN) are kinetically neutral below 100 °C, but at 140 °C, these compounds undergo decomposition to sulfenic acids and/or methylsulfinyl radicals, species able to trap lipidperoxyl radicals, resulting in better oxidative stability of the lipid system.

In the perspective (“Antioxidants, Food Processing and Health”), the authors discussed the loss and/or modification of antioxidants following the application of heat treatment, UV, pulsed electric field, drying, blanching, and irradiation [21]. Antioxidants mainly undergo oxidative phenomena and less pyrolysis and hydrolysis. These compounds are capable of other cell signaling, regulating the activities of transcription factors and determinants of gene expression.

In the review article titled “Impact of Emerging Technologies on Virgin Olive Oil Processing, Consumer Acceptance, and the Valorization of Olive Mill Wastes”, the authors collected and discussed the latest studies on the effect of emerging technologies (pulsed electric fields, high pressure, ultrasonic treatment) on EVOO quality [22]. Additionally, consumer acceptability of these new technological approaches was discussed. Finally, the impact of these innovative technologies in the valorization of EVOO processing waste was also addressed.

In the review article titled “Impact of Innovative Technologies on the Content of Vitamin C and Its Bioavailability from Processed Fruit and Vegetable Products”, Mieszczakowska-Frąc et al. [23] discussed the effects of modern fruit and vegetable processing technologies (high pressure processing, high pressure homogenization, minimal processing, ultrasound and electric fields pulsed) on the stability and bioavailability of vitamin C, one of the antioxidants in which consumers are particularly interested.

This Special Issue collected articles, perspectives, and reviews that unequivocally evidenced the importance of food processing in preserving nutrients and non-nutrients with reference to antioxidant phytochemicals. In conclusion, the food industries will have to adopt technological measures related to the maintenance of low temperatures and the application of pressures or pre-treatments and/or formulations to guarantee an aliquot of bioavailable antioxidant compounds.
